# Nonunion of the First Sternocostal Synchondrosis Accompanied by Sternoclavicular Joint Synovitis

**DOI:** 10.1155/2014/798329

**Published:** 2014-08-28

**Authors:** Makoto Takeuchi, Tomohiro Goto, Kiminori Yukata, Naoto Suzue, Daisuke Hamada, Toshihiko Nishisho, Ichiro Tonogai, Tetsuya Matsuura, Koichi Sairyo

**Affiliations:** Department of Orthopedics, Institute of Health Biosciences, The University of Tokushima Graduate School, 3-18-15 Kuramoto, Tokushima 770-8503, Japan

## Abstract

Injury to the sternocostal synchondrosis of the first rib is quite rare. We report one such case in a 50-year-old man with nonunion of the first sternocostal synchondrosis accompanied by synovitis of the sternoclavicular joint. He first underwent arthroscopic surgery of the left sternoclavicular joint. Postoperatively, the patient's symptoms decreased by half, but another pain and crepitus at the inferior lateral portion of the sternoclavicular joint developed. Since MRI and functional CT reexaminations revealed nonunion of the first sternocostal synchondrosis, resection arthroplasty of the first sternocostal joint was performed. This resulted in immediate resolution of the symptoms. At 2-year follow-up, his symptoms disappeared entirely with no limited range of motion of the shoulder.

## 1. Introduction

The most common site of first rib fracture is the middle-third of the diaphysis [1], because the groove for the subclavian artery (subclavian sulcus) is thin and therefore a weak point [2]. The mechanism of first rib fracture is direct trauma to the sternum, clavicle, anterior chest wall, upper thorax, or shoulder girdle or indirect trauma due to a sudden strong contraction of the scalenus muscles [3, 4]. In some cases, isolated fractures might occur as stress fractures attributed to repeated muscle contraction [5, 6]. On the other hand, injury to the sternocostal synchondrosis of the first rib is quite rare [7, 8]. Here, we present a case of nonunion of the first sternocostal synchondrosis accompanied by synovitis of the sternoclavicular joint.

## 2. Case Report

**Figure 1:**
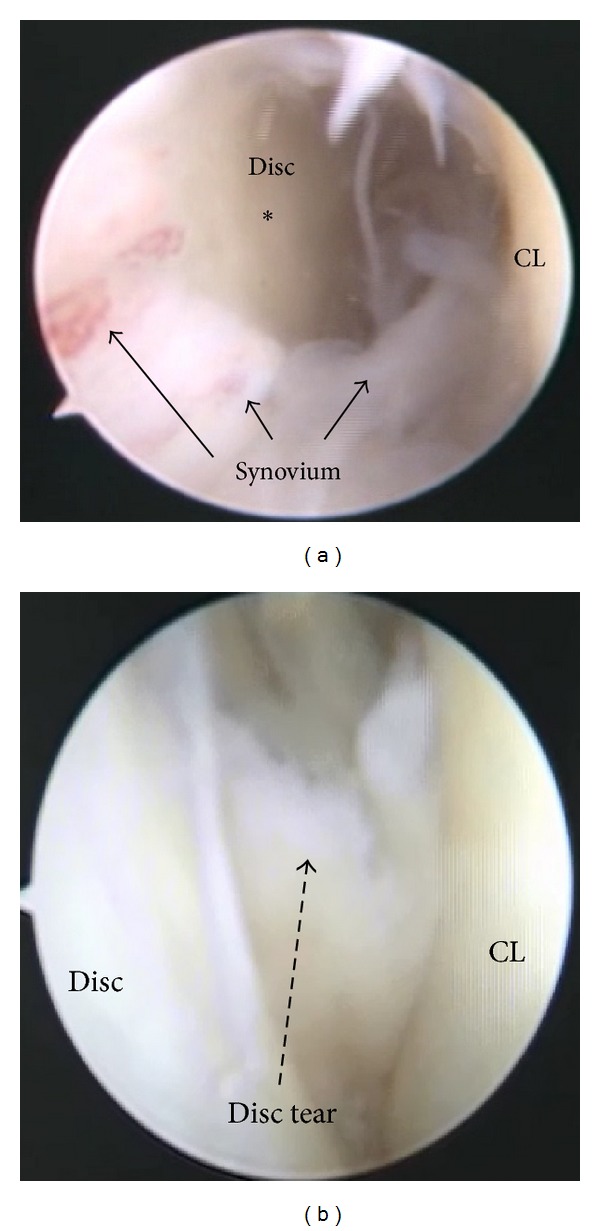
Arthroscopic images of the left sternoclavicular joint. (a) Inferior portal view showing synovial proliferation in the sternoclavicular joint (black arrows: synovium; a dot mark: intra-articular disc; CL: joint surface of the clavicle). (b) Inferior portal view showing a partial tear at the anterior part (a dotted arrow) of the intra-articular disc.

**Figure 2:**
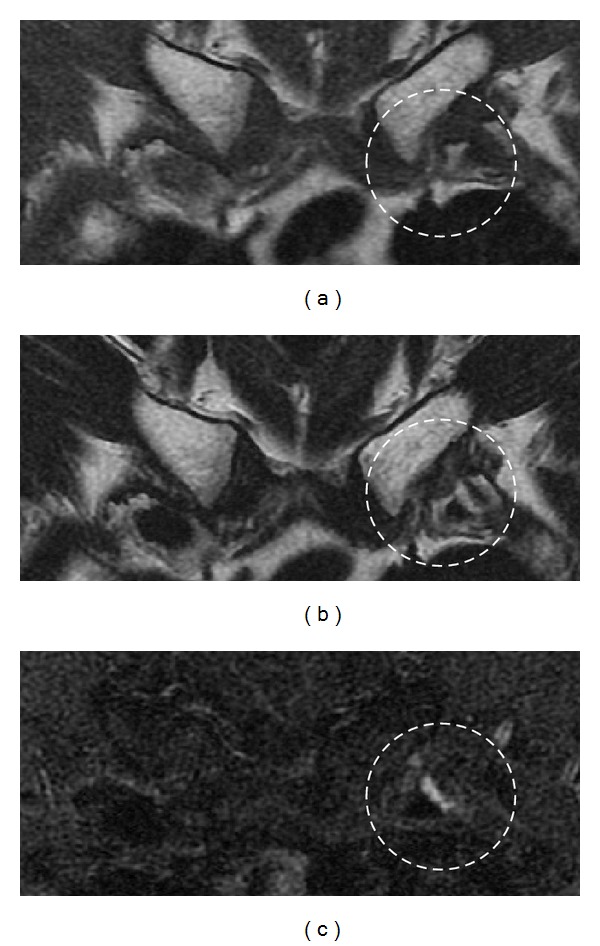
Magnetic resonance imaging of the bilateral first sternocostal synchondrosis. Coronal T1-weighted image (a), T2-weighted image (b), and STIR image (c) are shown. STIR image showing a high-intensity lesion at the left first sternocostal synchondrosis.

**Figure 3:**
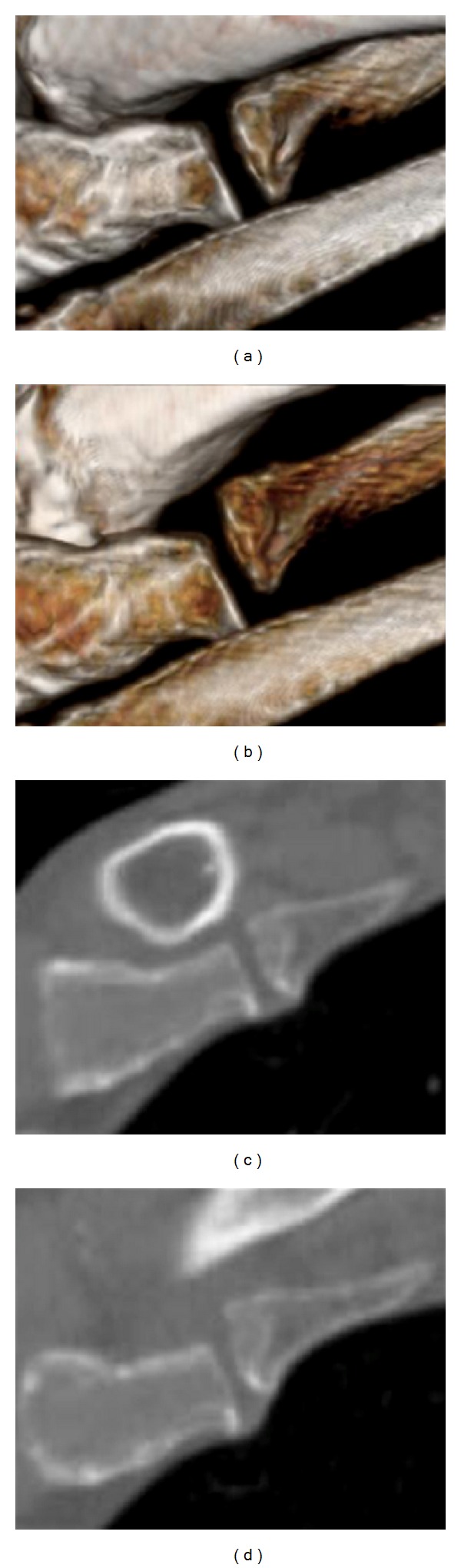
Computed tomography (CT) images of the left first sternocostal synchondrosis. Three-dimensional reconstruction (a, b) and coronal CT images (c, d) showing subluxation of the left first rib in two different positions, with the arm hanging naturally (a, c) and elevated (b, d).

A 50-year-old, right-hand-dominant man presented with left upper thoracic pain upon shoulder elevation. One year earlier, he was involved in a head-on car collision, which resulted in acute pain in the upper half of the body when moving his left arm forward. He was seated in the front right (driver's) seat and wearing a three-point seatbelt at the time of the accident. Cervical spine sprain and left shoulder contusion were diagnosed at an emergency hospital, and although he was treated with medication and rehabilitation, his symptoms did not improve. Physical examination of the left glenohumeral joint revealed no limited range of motion, muscle weakness, or signs of instability, but tenderness was noted around the sternoclavicular joint. Crepitus and severe pain in the left upper thorax occurred during moving the left shoulder downward from 80 to 70 degrees in flexion. A lower brachial plexus injury was also suspected because of a sensory disturbance in the C8 cervical root area and weak grip (10 kg; measured using a squeeze dynamometer). Although initial examination of the sternoclavicular joint on plain radiography, computed tomography (CT), and magnetic resonance imaging (MRI) revealed no obvious abnormalities, injection of corticosteroid and local anesthetic into the sternoclavicular joint temporally reduced the pain. As we assumed that his symptoms were caused by injury to the sternoclavicular joint disc accompanied by synovitis, we performed arthroscopic surgery of the left sternoclavicular joint. Because arthroscopic findings demonstrated synovitis and partial tear of the intra-articular disc of the left sternoclavicular joint (Figure 1), synovectomy and partial disc excision were performed. Postoperatively, the patient's symptoms decreased by half, but another pain and crepitus at the inferior lateral portion of the sternoclavicular joint developed a few days after the initial surgery. Careful MRI reexamination revealed a high-signal intensity lesion on short time inversion recovery images of the left first sternocostal synchondrosis (Figure 2). Then, multiplaner CT of the shoulder in two positions (0 degrees flexed position and elevated position) revealed separation and abnormal mobility at the left first rib synchondrosis (Figure 3). We finally diagnosed nonunion of the first sternocostal synchondrosis based on the finding that the pain disappeared immediately and completely after injecting corticosteroid and local anesthetic into the nonunion site under fluoroscopy.

The patient was treated conservatively with corticosteroid injection for 4 months, but his symptoms persisted. We, therefore, performed resection arthroplasty for nonunion of the first rib synchondrosis. Intraoperatively, subluxation of the first rib was observed upon flexion of the left upper arm. Dissection of the subclavius muscle and costoclavicular ligaments from the left clavicle improved the abnormal mobility of the first rib. Both cartilage surfaces of the nonunion site were irregular, and both the sternum and the first rib showed degeneration. The medial end of the first rib was removed until no contact with the sternum was obtained. Postoperatively, the pain and crepitus in the left upper thorax disappeared entirely, with no limited range of motion of the shoulder or recurrence of symptoms noted at the 2-year follow-up.

## 3. Discussion

Injury to the first costochondral joint is relatively uncommon compared with injury to lower costochondral joints. To our knowledge, only a few cases of injury to the first sternocostal synchondrosis have been reported [7, 8]. One of these cases showed nonunion similar to the present case, but no specific etiology was mentioned. Injury to the sternocostal synchondrosis might be difficult to detect at the initial examination because of its cartilaginous composition, which cannot be identified on radiography or CT.

The mechanism of first rib injury in car collisions is thought to be violent contraction of the scalene muscles brought about by the sudden forward movement of the head and neck and, in a car collision, consequent sudden arrest by the seatbelt [8, 9]. Another possible cause of this type of fracture is inflation of the driver's airbag [10]. The patient experienced a head-on car collision while wearing a three-point seatbelt in the present case. However, we also considered another possible injury mechanism. At the time of injury, the patient's left arm was jolted violently forward, which likely resulted in lower brachial plexus injury since the sternum was fixed in place by the three-point seat belt. From a kinematic point of view and due to the unique anatomic interrelationship between the first rib, manubrium, and clavicle, the collision might have generated an anterior force on the first rib through the subclavius muscle and costoclavicular ligaments, which connect the medial clavicle to the synchondral junction of the first rib, resulting in the left clavicle jolting forward and rotating anteriorly when the left arm was moved forward [11–13]. Disappearance of the abnormal mobility of the first rib after surgical resection of the subclavius muscle and costoclavicular ligament supports this speculation.

The patient was first diagnosed as having a disc injury accompanied by synovitis of the sternoclavicular joint for which he underwent arthroscopic partial discectomy and synovectomy of the left sternoclavicular joint. His symptoms immediately improved by half. Based on this observation, the synovitis could have been secondary to the instability due to nonunion of the first sternocostal synchondrosis, since the clavicle, manubrium, and the first costal cartilage constitute the joint's articulation through being connected by the intra-articular ligament [12, 13]. Some authors have reported that CT is useful for diagnosing first rib injury [11, 14, 15]. The costal cartilage of the first rib anatomically fuses directly to the manubrium to form synchondrosis [13]. Previous cases of the first sternocostal injury have had a small bone fragment at the fracture site, whereas the present injury was purely a cartilage lesion of the first sternocostal synchondrosis with no bone fragments and displacement of the nonunion site was limited when the arm was hanging naturally. Therefore, no abnormalities could be detected on plain CT or radiography. MRI with STIR sequences was useful for detecting abnormal effusion at the nonunion site. Moreover, comparative evaluation of the CT images taken in two different arm positions provided valuable information that revealed the instability of the first sternocostal synchondrosis.

Conservative therapies, such as medication, rest, and rehabilitation, are recommended as initial treatments for first rib injury. When conservative treatment is not successful, resection arthroplasty for first rib nonunion is effective for relieving symptoms [16–18]. In the present case, resection arthroplasty for nonunion of the first sternocostal synchondrosis was effective for complete resolution of severe pain and crepitus. Other surgical options such as costochondral joint fixation or dissection of the subclavius muscle and costoclavicular ligament are potential indications for this type of case. Costochondral joint fixation is a more complicated procedure with potentially fatal complications such as pneumothorax, nerve injury, and vascular injury. Moreover, relatively careful rehabilitations and shoulder range of motion restrictions may be needed postoperatively. According to the intraoperative findings, instability of the first rib disappeared after dissection of the subclavius muscle and costoclavicular ligament, but we observed these findings only under general anesthesia with muscle relaxant. Therefore, only dissection of these tissues seemed difficult in anticipation of successful treatment.

In summary, physicians should pay careful attention to injury of the first costochondral joint when a patient presents with persistent pain in the sternoclavicular joint after a head-on car collision. MR fat suppression imaging and/or dynamic CT evaluation in different arm positions can provide critical information for precisely diagnosing sternocostal synchondrosis injury.
